# The Efficacy and Safety of Pirfenidone Combined With Immunosuppressant Therapy in Connective Tissue Disease-Associated Interstitial Lung Disease: A 24-Week Prospective Controlled Cohort Study

**DOI:** 10.3389/fmed.2022.871861

**Published:** 2022-05-12

**Authors:** Jiaqi Wang, Xiao Wang, Xiaoyan Qi, Zhijian Sun, Tao Zhang, Yi Cui, Qiang Shu

**Affiliations:** ^1^Department of Rheumatology, Qilu Hospital, Cheeloo College of Medicine, Shandong University, Jinan, China; ^2^Shandong Provincial Clinical Research Center for Immune Diseases and Gout, Jinan, China; ^3^Department of Biostatistics, School of Public Health, Cheeloo College of Medicine, Shandong University, Jinan, China; ^4^Department of Radiology, Qilu Hospital, Cheeloo College of Medicine, Shandong University, Jinan, China

**Keywords:** connective tissue disease, interstitial lung disease, pirfenidone (PFD), systemic sclerosis, inflammatory myopathy, rheumatoid arthritis

## Abstract

**Objective:**

Interstitial lung disease (ILD) is a common manifestation of connective tissue disease (CTD) that manifests as several subtypes with significant differences in prognosis. It is necessary to evaluate the efficacy and safety of pirfenidone (PFD) combined with immunosuppressant (IS) in the treatment of CTD-ILD.

**Methods:**

A total of 111 patients with CTD-ILD were enrolled, including those with systemic sclerosis (SSc), inflammatory myopathy (IIM), rheumatoid arthritis (RA), and other CTDs (such as systemic lupus erythematosus, primary Sjogren's syndrome, and undifferentiated CTD). After evaluation of the high-resolution computed tomography (HRCT), pulmonary function (PF), and basic disease activity, patients either were or were not prescribed PFD and were followed up regularly for 24 weeks.

**Results:**

After 24 weeks of treatment, predicted forced vital capacity (FVC%) in the SSc-PFD group had improved by 6.60%, whereas this value was 0.55% in patients with SSc-no-PFD. The elevation in FVC% was also significant in IIM-PFD over the IIM-no-PFD controls (7.50 vs. 1.00%). The predicted diffusing capacity for carbon monoxide (DLCo%) of RA-PFD was enhanced by 7.40%, whereas that of RA-no-PFD decreased by 5.50%. When performing a subtype analysis of HRCT images, the change in FVC% among patients with SSc with a tendency toward usual interstitial pneumonia (UIP) was higher in those given PFD (SSc-PFD-UIP) than the no-PFD group (8.05 vs. −3.20%). However, in IIM patients with a non-UIP tendency, PFD displayed better therapeutic effects than the control (10.50 vs. 1.00%). DLCo% improved significantly in patients with the PFD-treated RA-non-UIP subtype compared with the patients with no-PFD (10.40 vs. −4.45%). Dichotomizing the patients around a baseline FVC% or DLCo% value of 70%, the PFD arm had a more improved FVC% than the no-PFD arm within the high-baseline-FVC% subgroups of patients with SSc and IIM (6.60 vs. 0.10%, 6.30 vs. 1.10%). In patients with RA-PFD, DLCo% showed a significant increase in the subgroup with low baseline DLCo% compared to that in patients with RA-no-PFD (7.40 vs. −6.60%).

**Conclusion:**

The response of PF to PFD varied between CTD-ILD subsets. Patients with SSc and IIM showed obvious improvements in FVC%, especially patients with SSc-UIP and IIM-non-UIP. In RA, the subsets of patients with non-UIP and a lower baseline DLCo% most benefited from PFD.

## Introduction

Connective tissue disease (CTD), including systemic sclerosis (SSc), inflammatory myopathy (IIM), rheumatoid arthritis (RA), systemic lupus erythematosus (SLE), and primary Sjogren's syndrome (pSS), is a cluster of autoimmune diseases with multiorgan involvement. The lung is one of the most commonly affected organs. There are different subtypes of pathology and imaging manifestations of CTD-associated interstitial lung disease (ILD), and these patients may present with subclinical features following a slow or acute progressive course, the latter showing clinically significant rapid progression and mortality.

The incidence of CTD-ILD is reported to range from 12.4 to 34% (1). The etiology and pathogenesis of CTD-ILD are still unclear, but immune-mediated pulmonary inflammation and subsequent fibrosis are key elements in the development of the condition (2).

Different CTDs can manifest as different or same types of ILD. Thus far, pathological examination is the gold standard for typing, and the high-resolution computed tomography (HRCT) findings correspond well with the pathological changes. The common types of CTD-ILD are non-specific interstitial pneumonia (NSIP), usual interstitial pneumonia (UIP), organizing pneumonia (OP), and lymphocytic interstitial pneumonia (LIP) (3–5).

In terms of CTD-ILD therapy, the Chinese guideline of 2018 emphasizes treatment of both CTD activity and ILD progression. Glucocorticoids (GCs) combined with immunosuppressive agents (IS) are used as the first-line treatment for different CTD-ILD, with no recommendations on how to use GCs for different imaging types, such as NSIP and UIP. Unfortunately, some patients have a poor response to such therapy due to fibrosis progression. Pirfenidone (PFD) is a pyridone-derived drug with extensive antifibrotic, anti-inflammatory, and antioxidant effects that modulates a number of cytokines, such as transforming growth factor-β1, interleukin (IL)-1, IL-4, IL-6, IL-8, IL-13, and tumor necrosis factor-α (6–9). It is currently approved worldwide for the treatment of idiopathic pulmonary fibrosis (IPF) based on its ability to slow the pulmonary function (PF) decline and disease progression, as shown in a number of phase III clinical trials (10–12). Based on these findings, PFD has been applied for some kinds of CTD-ILD, but few randomized clinical trials or strictly controlled studies have been reported (13, 14).

To verify whether PFD is effective for CTD-ILDs, our study was conducted to observe the efficacy and safety of PFD combined with GC and IS for the treatment of CTD-ILD.

## Materials and Methods

### Patients

A total of 177 patients who met the diagnostic criteria of CTD-ILD were treated from August 2019 to May 2021 at the Department of Rheumatology of Qilu Hospital, Shandong University. All participants fulfilled the following criteria, namely, (1) age ≥ 18 years and (2) meeting the international classification standard of a CTD, including SSc, IIM, RA, SLE, pSS, and undifferentiated CTD (UCTD) (15–19). The ILD diagnosis conformed to the criteria of HRCT and PF formulated by the American Thoracic Association and the European Respiratory Association in 2002 (20). Detailed descriptions of the exclusion criteria are provided in Supplementary Appendix S1.

### Study Design

In this prospective, controlled, single-center study, patients with CTD-ILD had all received a stable dosage of GC and/or IS as background therapy since 4 weeks before baseline and were followed up regularly for 24 weeks. The dosage of combined GC and the kind of IS was determined from the clinical characteristics of the different CTDs and was maintained throughout the study, regardless of antifibrosis. After the evaluation of PF (FVC% and DLCo%), HRCT, and basic disease activity, physicians recommended whether to add PFD and solicited the opinions of patients according to the inclusion criteria. PFD was initially prescribed at a 300 mg/day dosage, and the dosage was increased to the maximum tolerable dosage or to a maximum of 1,800 mg/day. The primary end point of our study was the change in PF after 24 weeks of treatment.

The prescribing principles of PFD in patients with CTD-ILD were (1) the patient fulfilled the inclusion and exclusion criteria of CTD-ILD; (2) the patient had a low FVC% and/or DLCo% value (usually <80%) or no PF improvement with GC and IS treatment in the past several months; and (3) the patient had symptoms of cough or dyspnea after activities, or the imaging area of fibrosis was large or tended to expand.

The reasons for non-use of PFD were (1) no significant deteriorations in respiratory symptoms, HRCT scan, or PF in the screening state; and (2) patients' will. In this real-world study, most patients with CTD-ILD were reluctant to use PFD unless they felt the disease was serious, mainly due to the price of PFD and their insurance status.

All participants were forewarned of potential photosensitivity manifesting as a skin rash and were advised to use sunscreen during exposure to direct sunlight. This investigation was reviewed by the Ethics Committee of Qilu Hospital, Shandong University, and conducted in compliance with the Declaration of Helsinki (KYLL-202008-014). The study was registered in the Clinical Trial Registry (NCT04928586).

### Study Assessments

Baseline data regarding the demographics, PF, HRCT, laboratory characteristics, disease activity (IIM, manual muscle test 8, myositis disease activity assessment visual analog scale, RA, disease activity score in 28 joints, clinical disease activity index, simplified disease activity index, and health assessment questionnaire) (21, 22), and previous therapy history of patients with CTD-ILD were collected. After 24 weeks of treatment, the above follow-up data and adverse events (AEs) were recorded in both the Qilu Hospital database and at the Chinese Rheumatism Data Center (CRDC) with the Chinese Rheumatology Information Platform (CRIP). The HRCT imaging characteristics were assessed independently by two experienced radiologists who were blinded to the final diagnosis of the lesions.

### Statistical Analysis

Continuous variables that conformed to a normal distribution are expressed as mean ± SD, whereas continuous variables that did not conform to a normal distribution are expressed as median and interquartile range. The change between the 24-week value and the baseline value was further calculated (value of change = value at 24 weeks – value at baseline). The differences in continuous variables between two groups were analyzed by the independent-sample *t*-test and the Wilcoxon rank-sum test, whereas those within groups were evaluated using the paired *t*-test or paired Wilcoxon test. The chi-squared test and Fisher's exact probability test were used to compare the rates. Multiple linear regression analysis was used to evaluate the factors influencing the changes in FVC% and DLCo%. A *p* < 0.05 was considered statistically significant. All analyses were performed using the GraphPad Prism 8 and SPSS 24 software (IBM, Armonk, NY, USA).

## Results

### Baseline Characteristics of Patients

**Figure 1:**
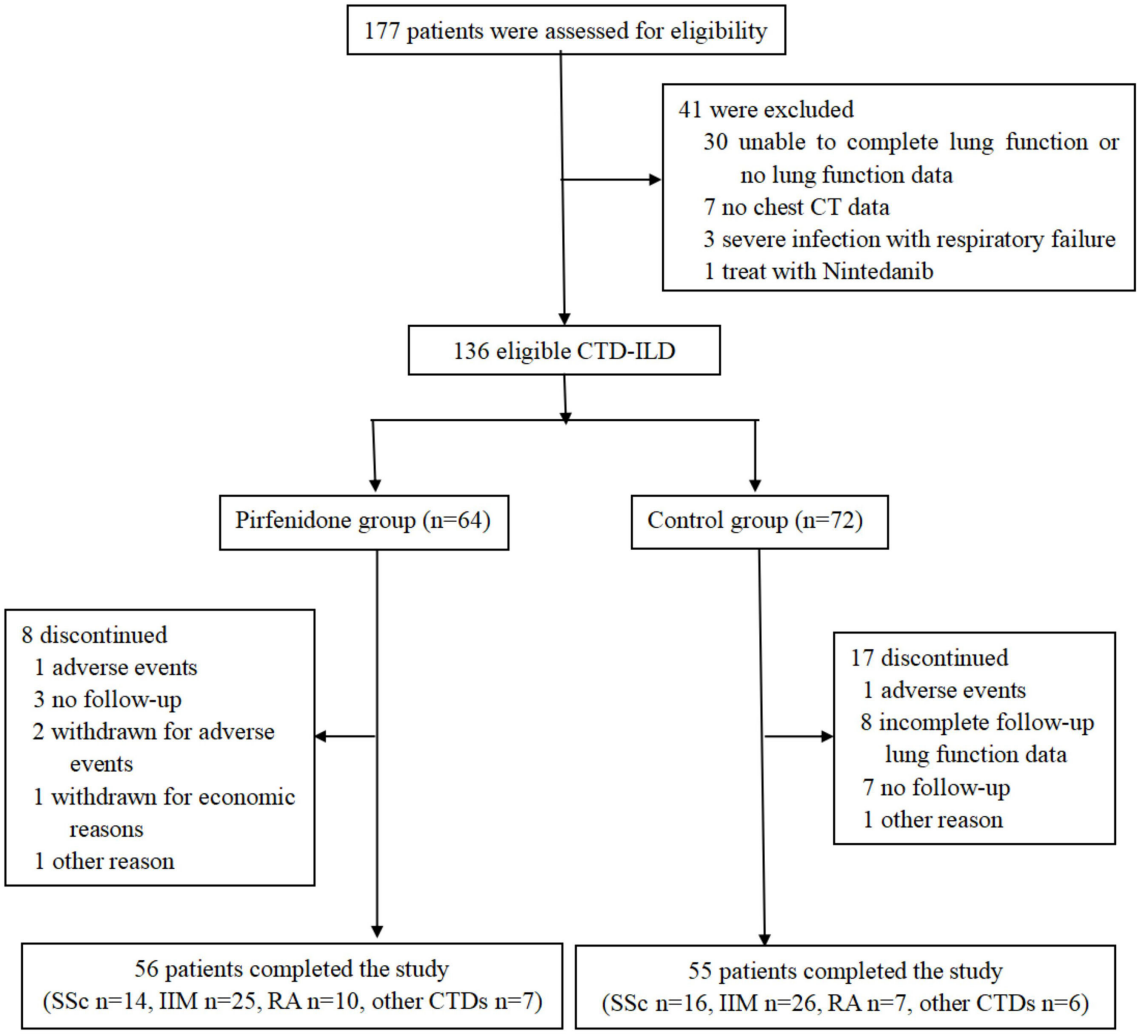
Flowchart of this study.

The screening process of 177 patients is illustrated in Figure 1. A total of 136 patients were eligible after the evaluation of PF, HRCT, and basic disease. Then, 64 patients were prescribed PFD, and 72 patients were not (control group), all of whom were followed up regularly. Eventually, 111 patients completed the 24-week observation and were included in the analysis (56 in the PFD group, 55 in the control group).

We categorized the patients with CTD-ILD into 4 disease groups, namely, SSc, IIM, RA, and other CTDs (including SLE, pSS, and UCTD). The demographic and clinical characteristics, PF, HRCT imaging, and therapeutic regimen at baseline are shown in Table 1.

**Table 1:** The baseline clinical characteristics, PF, HRCT imaging features, and therapeutic regimen of the 4 CTD-ILD groups.

	**SSc (*n* = 30)**	**IIM (*n* = 51)**	**RA (*n* = 17)**	**Other CTDs (*n* = 13)**	***p* value**
Age-years	45.17 ± 12.96	50.75 ± 10.57	56.12 ± 11.87	53.23 ± 10.73	**0.013**
Females (%)	29 (97.0)	39 (76.0)	13 (76.0)	12 (92.0)	**0.050**
BMI (kg/m^2^)	23.16 ± 3.77	23.93 ± 2.92	25.36 ± 3.10	23.23 ± 3.34	0.143
Former smoker (%)	4 (13.0)	4 (8.0)	3 (18.0)	1 (8.0)	0.611
Disease course (months)	24.00 (11.25–55.75)	7.50 (1.00–17.00)	60.00 (4.50–95.50)	31.00 (1.75–111.00)	**0.003**
FVC%	90.97 ± 21.17	84.8 ± 18.19	87.43 ± 16.16	87.08 ± 20.00	0.574
DLCo%	65.55 ± 18.34	68.71 ± 14.4	66.89 ± 12.17	54.58 ± 15.25	**0.036**
FVC%<70%	4 (13.3)	15 (29.4)	2 (11.8)	2 (15.4)	0.269
DLCo%<70%	16 (57.1)	28 (56.0)	10 (58.8)	12 (92.3)	0.064
Activity-related dyspnea (%)	21 (70.0)	33 (65.0)	9 (53.0)	6 (46.0)	0.401
Unusual physical signs (%)	8 (27.0)	14 (27.0)	4 (24.0)	3 (23.0)	1.000
**Thoracic HRCT scan (%)**					**0.01**
UIP	3 (10.0)	6 (12.0)	7 (44.0)	1 (8.0)	
NSIP	27 (90.0)	42 (82.0)	7 (44.0)	12 (92.0)	
OP	0 (0.0)	3 (6.0)	1 (6.0)	0 (0.0)	
LIP	0 (0.0)	0 (0.0)	1 (6.0)	0 (0.0)	
UIP tendency on HRCT (%)	11 (36.7)	17 (33.3)	9 (52.9)	4 (30.8)	0.501
ESR (mm/h)	35.50 (17.75–55.75)	18.00 (8.25–35.75)	50.00 (29.50–86.50)	28.50 (9.25–48.25)	**0.005**
CRP (mg/L)	0.80 (0.37–2.72)	0.62 (0.22–5.10)	6.49 (3.40–18.00)	1.46 (0.54–2.72)	**0.002**
Hemoglobin (g/L)	127.50 (116.50–137.75)	135.50 (127.30–146.00)	134.00 (117.00–142.00)	132.50 (117.00–143.50)	0.149
Albumin (g/L)	46.05 (42.48–48.10)	43.30 (38.08–45.98)	41.60 (37.60–44.85)	44.45 (42.43–48.18)	**0.011**
Globulin (g/L)	30.85 (28.75–33.63)	26.00 (22.75–30.85)	30.30 (25.75–33.20)	29.70 (25.20–37.35)	0.059
**Baseline treatment**					
GC use (%)	27 (90.0)	51 (100.0)	16 (94.1)	12 (92.3)	0.607
GC dosage (mg/d prednisone)	10.00 (5.00–15.00)	20.0 (12.5–45.00)	12.50 (7.50–20.00)	7.50 (2.80–20.00)	<0.001
HCQ use (%)	25 (83.3)	36 (70.6)	12 (70.6)	10 (76.9)	0.608
Present DMARDs (%)					<0.001
None	3 (10.0)	6 (11.8)	1 (5.9)	2 (15.4)	
MMF	16 (53.3)	12 (23.5)	0 (0.0)	7 (53.8)	
TAC	1 (3.3)	24 (47.1)	8 (41.7)	1 (7.7)	
JAKi	9 (30.0)	6 (11.8)	4 (23.5)	0 (0.0)	
Others	1 (3.3)	3 (5.9)	4 (23.5)	3 (23.1)	
**Previous treatment**					
GC use (%)	27 (90.0)	51 (100.0)	15 (88.2)	12 (92.3)	**0.037**
HCQ use (%)	23 (76.7)	36 (70.6)	11 (64.7)	11 (84.6)	0.628
DMARDs use (%)	25 (86.2)	45 (88.2)	15 (88.2)	12 (92.3)	1.000

*PF, pulmonary function; other CTDs: included SLE, pSS, and UCTD; unusual physical signs: included cyanosis, velcro rale, and clubfoot; UIP, usual interstitial pneumonia; NSIP, nonspecific interstitial pneumonia; LIP, lymphocytic interstitial pneumonia; OP, organizing pneumonia; UIP tendency on HRCT: includes a definite UIP pattern and probable UIP pattern expressed by reticulation and honeycombing; GC, glucocorticoids; HCQ, hydroxychloroquine; DMARDs, disease-modifying antirheumatic drugs; MMF, mycophenolate mofetil; TAC, tacrolimus; JAKi, JAK inhibitor; others: other immunosuppressive drugs including iguratimod, cyclophosphamide, and cyclosporine*.

*Age, BMI, and baseline PF data are presented as means and standard deviations and tested by the t-test. The other data are presented as medians and ranges and were tested by the Mann-Whitney U-test. Bold numbers denote statistical significance*.

Among the four groups, patients with RA-ILD were older, had a longer disease duration, had higher ESR and CRP levels, and had lower serum albumin than the other groups (all *p* < 0.05). The SSc-ILD group was the youngest, and the patients with IIM-ILD had the shortest disease duration between the 4 groups (all *p* < 0.05). As shown in PF, the DLCo% in the other CTDs was lower than that of the SSc, IIM, and RA groups (54.58% ± 15.25% vs. 65.55% ± 18.34%, 68.71% ± 14.4%, 66.89% ± 12.17%, *p* = 0.036). In the field of HRCT imaging, a definite UIP pattern was observed in 7 (44%) patients with RA-ILD, which was more prevalent than that in the other three groups (*p* = 0.010). There were no significant differences in baseline FVC%, activity-related dyspnea, or unusual physical signs between the 4 groups.

In terms of the background treatment at baseline, the individuals with IIM-ILD were more likely to take high-dosage GC (*p* < 0.001). With regard to IS, patients with SSc-ILD were more likely to receive mycophenolate mofetil (MMF) and JAK inhibitor (JAKi). Tacrolimus (TAC) and MMF were frequently used in patients with IIM-ILD, whereas patients with RA-ILD preferred TAC and JAKi. The patients in the other CTDs group were more likely to receive MMF. There were no differences in UIP tendency, previous use of GC, hydroxychloroquine (HCQ), or IS between the 4 CTD-ILD groups.

Both baseline FVC% and DLCo% in the PFD group were lower than those in the control group (*p* < 0.001 and *p* = 0.005). Simultaneously, the individuals in the PFD group were more likely to have a high UIP tendency (*p* = 0.002). The baseline FVC% of patients with SSc-PFD and IIM-PFD was lower than that of patients with SSc-no-PFD and IIM-no-PFD, respectively (*p* = 0.014 and *p* = 0.010). Patients in the IIM group who received PFD generally had a low baseline DLCo% and high UIP tendency on HRCT (*p* = 0.034 and 0.008, respectively). There were no differences in the GC dosage or IS between the PFD and control groups in any of the 4 diseases. In addition, no differences were observed in the PF or HRCT scan type across the PFD groups of the 4 diseases (Tables 2A,B).

**Table 2A:** The baseline PF and HRCT imaging in PFD-treated and control groups of patients with CTD-ILD.

	**Total**		**SSc-ILD**		**IIM-ILD**		**RA-ILD**		**Other CTDs**	
	**Pirfenidone**	**Control**	**Pirfenidone**	**Control**	**Pirfenidone**	**Control**	**Pirfenidone**	**Control**	**Pirfenidone**	**Control**
	**(*n* = 56)**	**(*n* = 55)**	**(*n* = 14)**	**(*n* = 16)**	**(*n* = 25)**	**(*n* = 26)**	**(*n* = 10)**	**(*n* = 7)**	**(*n* = 7)**	**(*n* = 6)**
FVC%	80.58 ± 17.19^***^	93.81 ± 18.32	81.06 ± 18.81^*^	99.63 ± 19.69	78.23 ± 17.91^*^	91.12 ± 16.41	84.28 ± 15.60	91.93 ± 17.07	82.71 ± 15.60	92.17 ± 24.71
DLCo%	61.68 ± 13.40^**^	70.04 ± 16.88	60.65 ± 14.47	69.53 ± 20.57	64.25 ± 12.91^*^	72.82 ± 14.71	65.07 ± 9.57	69.49 ± 15.62	49.90 ± 13.47	60.03 ± 16.55
FVC%<70%	16 (28.6)	7 (12.7)	3 (21.4)	1 (6.3)	10 (40.0)	5 (19.2)	2 (20.0)	0 (0.0)	1 (14.3)	1 (16.7)
DLCo%<70	38 (70.4)^**^	28 (50.9)	10 (76.9)	6 (37.5)	15 (62.5)	13 (50.0)	6 (60.0)	4 (57.1)	7 (100.0)	5 (83.3)
Activity-related dyspnea (%)	36 (64.3)	33 (60.0)	11 (78.6)	10 (62.5)	18 (72.0)	15 (57.7)	5 (50.0)	4 (57.1)	2 (28.6)	4 (66.7)
Unusual physical signs (%)	16 (28.6)	13 (23.6)	3 (26.7)	5 (31.3)	10 (40.0)	4 (15.4)	1 (10.0)	3 (42.9)	2 (28.6)	1 (16.7)
Thoracic HRCT scan (%)										
UIP	12 (21.8)	5 (9.1)	3 (21.4)	0 (0.0)	5 (20.0)	1 (3.8)	3 (33.3)	4 (57.1)	1 (14.3)	0 (0.0)
NSIP	41 (74.5)	47 (85.5)	11 (78.6)	16 (100.0)	19 (76.0)	23 (88.5)	5 (55.6)	2 (28.6)	6 (85.7)	6 (100.0)
OP	2 (3.6)	2 (3.6)	0 (0.0)	0 (0.0)	1 (4.0)	2 (7.7)	1 (11.1)	0 (0.0)	0 (0.0)	0 (0.0)
LIP	0 (0.0)	1 (1.8)	0 (0.0)	0 (0.0)	0 (0.0)	0 (0.0)	0 (0.0)	1 (14.3)	0 (0.0)	0 (0.0)
UIP tendency on HRCT (%)	29 (51.8)^**^	12 (21.8)	8 (57.1)	3 (18.8)	13 (52.0)^**^	4 (15.4)	4 (40.0)	5 (71.4)	4 (57.1)	0 (0.0)

**Table 2B:** The baseline therapeutic regimen in PFD-treated and control groups of patients with CTD-ILD.

	**Total**		**SSc-ILD**		**IIM-ILD**		**RA-ILD**		**Other CTDs**	
	**Pirfenidone**	**Control**	**Pirfenidone**	**Control**	**Pirfenidone**	**Control**	**Pirfenidone**	**Control**	**Pirfenidone**	**Control**
	**(*n* = 56)**	**(*n* = 55)**	**(*n* = 14)**	**(*n* = 16)**	**(*n* = 25)**	**(*n* = 26)**	**(*n* = 10)**	**(*n* = 7)**	**(*n* = 7)**	**(*n* = 6)**
GC use (%)	32 (94.6)	53 (96.4)	13 (92.9)	14 (87.5)	25 (100.0)	26 (100.0)	9 (90.0)	7 (100.0)	6 (85.7)	6 (100.0)
GC (mg/d prednisone)	15.00 (5.00–32.50)	15.00 (7.50–20.00)	6.25 (3.75–22.50)	10.00 (6.88–15.00)	25.00 (15.00–42.50)	16.25 (12.50–50.00)	15.00 (8.75–25.00)	10.00 (7.50–20.00)	5.00 (3.44–13.75)	17.50 (2.23–30.00)
HCQ use (%)	37 (66.1)^*^	46 (83.6)	10 (71.4)	15 (93.8)	16 (64.0)	20 (76.9)	6 (60.0)	6 (85.7)	5 (71.4)	5 (83.3)
DMARDs (%)										
None	8 (14.3)	4 (7.3)	2 (14.3)	1 (6.3)	4 (16.0)	2 (7.7)	1 (10.0)	0 (0.0)	1 (14.3)	1 (16.7)
MMF	18 (32.1)	17 (30.9)	5 (35.7)	11 (68.8)	8 (32.0)	4 (15.4)	0 (0.0)	0 (0.0)	5 (71.4)	2 (33.3)
TAC	12 (21.4)	22 (40)	0 (0.0)	1 (6.3)	7 (28.0)	17 (65.4)	5 (50.0)	3 (42.9)	0 (0.0)	1 (16.7)
JAKi	12 (21.4)	7 (12.7)	6 (42.9)	3 (18.8)	4 (16.0)	2 (7.7)	2 (20.0)	2 (28.6)	0 (0.0)	0 (0.0)
Others	6 (10.7)	5 (9.1)	1 (7.1)	0 (0.0)	2 (8.0)	1 (3.8)	2 (20.0)	2 (28.6)	1 (14.3)	2 (33.3)

*PF, pulmonary function; other CTDs: included SLE, pSS, and UCTD; unusual physical signs: included cyanosis, velcro rale, and clubfoot; UIP, usual interstitial pneumonia; NSIP, non-specific interstitial pneumonia; LIP, lymphocytic interstitial pneumonia; OP, organizing pneumonia; UIP tendency on HRCT: includes definite UIP pattern and probable UIP pattern expressed by reticulation and honeycombing. GC, glucocorticoids; HCQ, hydroxychloroquine; DMARDs, disease-modifying antirheumatic drugs; MMF, mycophenolate mofetil; TAC, tacrolimus; JAKi, JAK inhibitor; others: other immunosuppressive drugs include iguratimod, cyclophosphamide, and cyclosporine*.

*Baseline pulmonary function (PF) data are presented as means and standard deviations and were tested by Student's t-test. The others are presented as medians and ranges and tested by Mann-Whitney U-test.*

* *p < 0.05*

**p < 0.01

***p < 0.001 compared to the control group.

All patients with CTD-ILD were relatively stable in extrapulmonary performance throughout the study. The classification and disease activity of these patients are detailed in Supplementary Tables 1–3.

### Changes in PF

**Figure 2:**
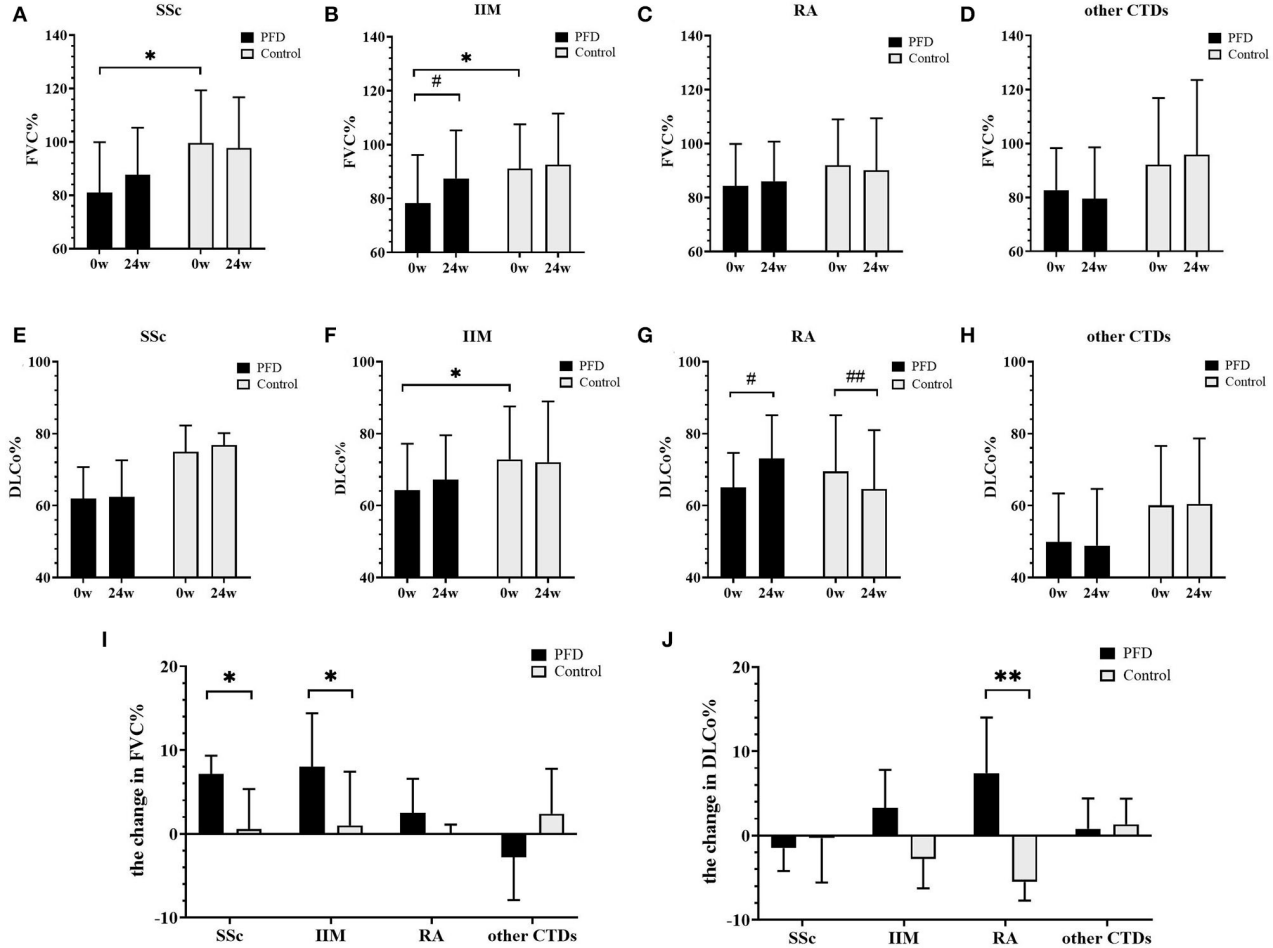
Changes in FVC% and DLCo% in the PFD-treated and control groups of patients with CTD-ILD over 24 weeks. FVC% changes in patients with **(A)** SSc-ILD, **(B)** IIM-ILD, **(C)** RA-ILD, and **(D)** other patients with CTD from baseline to 24 weeks. DLCo% changes in patients with **(E)** SSc-ILD, **(F)** IIM-ILD, **(G)** RA-ILD, and **(H)** other patients with CTD from baseline to 24 weeks. The changed value of **(I)** FVC% and **(J)** DLCo% in patients with SSc, IIM, RA, and other patients with CTD. *p < 0.05, **p < 0.01 compared to the no-PFD control; ^#^*p* < 0.05, ^##^ *p* < 0.01 compared to the baseline value. Baseline and 24-week pulmonary function data are presented as the means and standard deviations and were tested by the unpaired *t*-test. The other results are presented as medians and ranges and were tested by the Mann-Whitney *U*-test.

The changes in FVC% (Figures 2A–D,I) and DLCo% (Figures 2E–H,J) from baseline to 24 weeks were compared in the PFD group and control group. We found that after 24 weeks of treatment FVC% in the SSc-PFD group was improved by 6.60% (3.10–8.46%), while this value was 0.55% (−6.80 to 5.35%) in the SSc-no-PFD group (*p* = 0.042). The elevation in FVC% was also different between the PFD and control groups of patients with IIM: 7.50% (0.55–14.45%) vs. 1.00% (−4.65 to 7.43%) (*p* = 0.016). In contrast, the DLCo% of RA-PFD was enhanced by 7.40% (2.18–14.00%) compared with the RA-no-PFD decrease of 5.50% (−7.70 to −1.00%) from baseline (*p* = 0.002). No significant improvement in either the FVC% or DLCo% of the PFD group was found in the other CTD group.

### Analysis of HRCT Subtype

**Figure 3:**
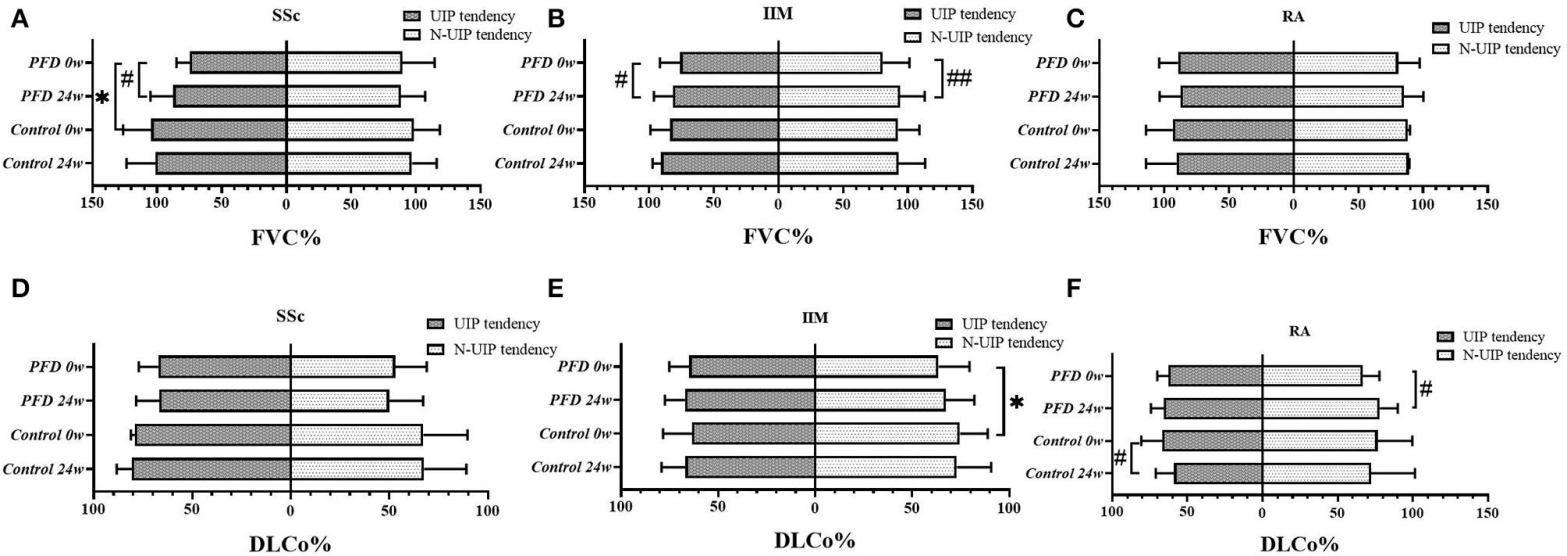
The PF value in different HRCT subtypes of the 3 CTD-ILD groups at baseline and 24 weeks comparing PFD treatment with the no-PFD control treatment. The value of FVC% in patients with **(A)** SSc-ILD, **(B)** IIM-ILD, and **(C)** RA-ILD and the value of DLCo% in patients with **(D)** SSc-ILD, **(E)** IIM-ILD, and **(F)** RA-ILD at baseline and 24 weeks comparing the efficacy of PFD in UIP and non-UIP tendency subtypes with that of the no-PFD control. *p < 0.05 compared to the no-PFD control; ^#^*p* < 0.05, ^##^*p* < 0.01 compared to baseline value.

When performing subtype analysis based on manifestations in HRCT, definite UIP and possible UIP patterns characterized by reticulation and/or honeycombing were grouped together as the UIP tendency in this study (23). Regarding the baseline FVC% of the SSc-UIP tendency subtype, the PFD-treated group had a lower value than the control (*p* = 0.030) (Figure 3A). A non-UIP tendency in patients with IIM was more likely to yield a poor DLCo% in the PFD group than in the control group (*p* = 0.036) (Figure 3E).

In addition, we found a significant improvement in FVC% from baseline to 24 weeks in the PFD group of SSc (*p* = 0.021, Figure 3A) and patients with IIM with UIP tendency (*p* = 0.027, Figure 3B). The same improvement in the FVC% of patients with IIM-non-UIP tendency was also detected after 24 weeks of PFD (*p* = 0.009, Figure 3B). DLCo% improved in patients with RA-non-UIP tendency after PFD treatment (*p* = 0.047, Figure 3F). There were no differences in the FVC% of patients with RA (Figure 3C) and DLCo% of patients with SSc (Figure 3D) in the PFD and control groups at either baseline or 24 weeks regardless of the HRCT subtypes.

**Figure 4:**
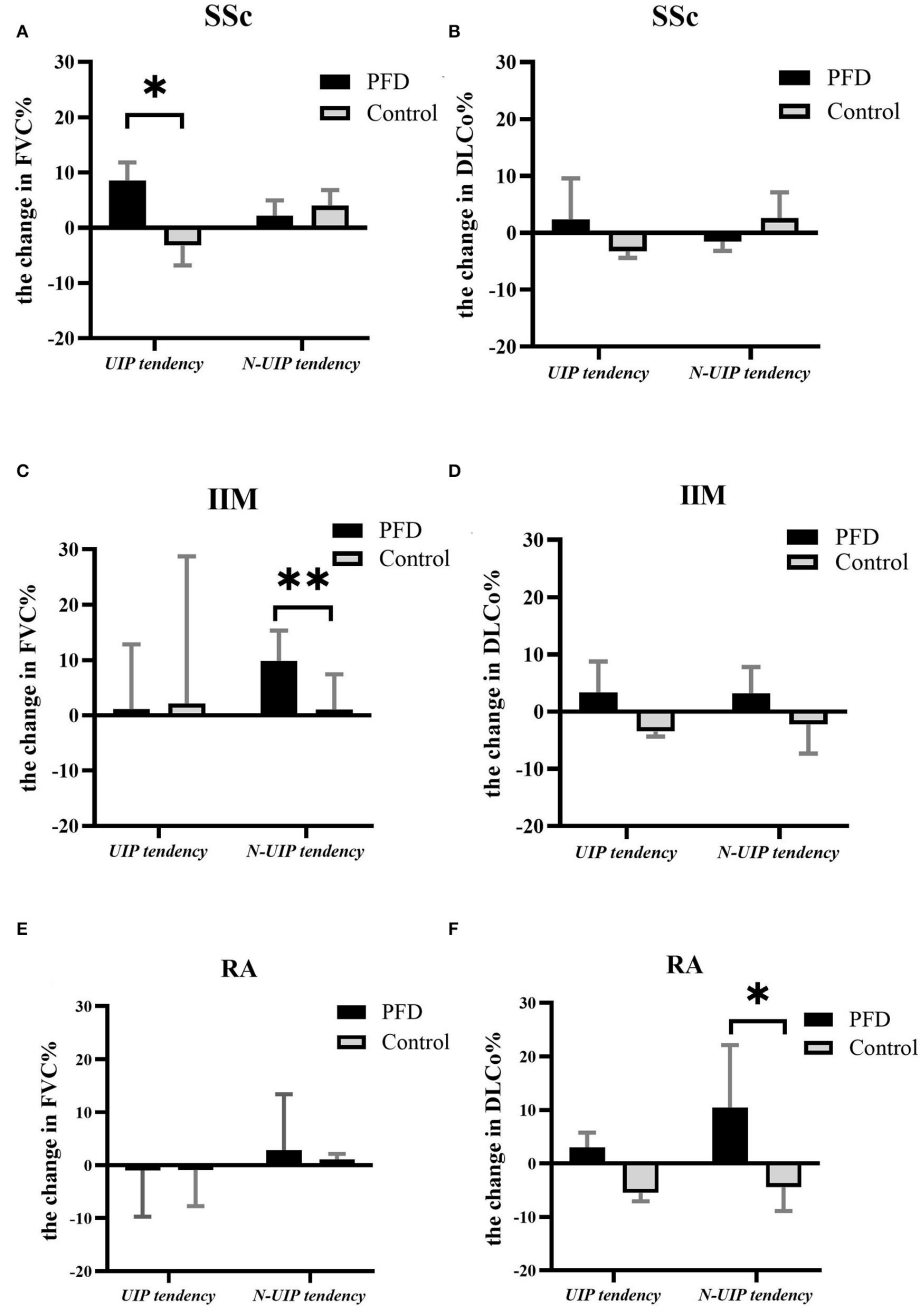
The change in PF in different HRCT subtypes of the 3 CTD-ILD groups with vs. without PFD. The change in FVC% in UIP and non-UIP tendency subtypes of patients with **(A)** SSc-ILD, **(C)** IIM-ILD, and **(E)** RA-ILD and the change in DLCo% in patients with **(B)** SSc-ILD, **(D)** IIM-ILD, and **(F)** RA-ILD either treated or not treated with PFD. *p < 0.05, **p < 0.01 compared to the no-PFD control.

After 24 weeks of treatment, the change in FVC% (Figures 4A,C,E) and DLCo% (Figures 4B,D,F) in different HRCT subtypes were compared between the PFD and control groups. We found the change in FVC% in patients with SSc-UIP given PFD was higher than in those not given PFD, 8.05% (6.15–19.43%) vs. −3.20% (−6.80 to 1.55%), *p* = 0.014 (Figure 4A). However, the non-UIP tendency subtype of patients with IIM given PFD showed a better change in FVC% than the patients with no-PFD: 10.50% (6.30–15.60%) vs. 1.00% (−4.65% to 7.43%), *p* = 0.005 (Figure 4C). DLCo% improved significantly in the PFD-treated RA-non-UIP subtype of patients than the patients with no-PFD: 10.40% (4.58–22.13%) vs. −4.45% (−8.90% to 2.1%), *p* = 0.017 (Figure 4F). There were no significant differences in GC or IS dosage at either baseline or follow-up between the different subgroups.

### Analysis of Baseline PF Subsets

We also classified patients in both the PFD intervention and control groups according to whether the baseline FVC% and DLCo% values were <70%. The results illustrated that the change in FVC% of the PFD group was higher than that of the control group in all patients with CTD-ILD regardless of whether the baseline FVC% was <70%. Particularly in the subset with baseline FVC% ≥ 70%, the PFD group exhibited an FVC% that was increased by 4.10% (−1.40 to 7.85%) compared with an increase of 0.30% (−3.10 to 4.68%) in the control group (*p* = 0.050) (Table 3).

**Table 3:** The change in FVC% and DLCo% between different baseline PF subsets of the three CTD-ILD groups, with vs. without PFD.

**Item**	**Category**	**Total**			**SSc-ILD**			**IIM-ILD**			**RA-ILD**		
	**Baseline PFT**	**Pirfenidone**	**Control**	***p***	**Pirfenidone**	**Control**	***p***	**Pirfenidone**	**Control**	***p***	**Pirfenidone**	**Control**	***p***
Change	FVC% ≥ 70%	4.10 (−1.40 to 7.85)	0.30 (−3.10 to 4.68)	0.050^*^	6.60 (1.23 to 11.50)	0.10 (−6.80 to 4.60)	0.047^*^	6.30 (0.50 to 10.50)	1.10 (−3.30 to 6.80)	0.089	0.00 (−5.98 to 4.85)	0.00 (−2.80 to 1.10)	0.643
in FVC%	(*n*)	40	48		11	15		15	21		8	7	
	FVC% ≥ 70%	10.88 (0.80 to 17.30)	1.00 (−7.20 to 14.20)	0.300	8.46 (3.80 to 8.54)	14.2 (–)	0.180	15.10 (0.35 to 19.10)	0.90 (−10.70 to 22.60)	0.221	9.85 (1.00 to 18.70)	–	–
	(*n*)	16	7		3	1		10	5		2	0	
Change	DLCo% ≥ 70%	−3.50 (−7.00 to 4.60)	−3.80 (−6.40 to 3.30)	0.969	−4.20 (−5.00 to 3.00)	−3.80 (−5.45 to 8.73)	0.519	−4.20 (−11.30 to 2.30)	−3.80 (−10.30 to 1.30)	0.815	6.25 (−3.75 to 18.95)	−5.00 (−6.40 to −2.50)	0.157
in DLCo%	(*n*)	16	27		3	10		9	13		4	3	
	DLCo% ≥ 70%	4.40 (−1.80 to 8.45)	−0.50 (−5.50 to 5.53)	0.057	−0.40 (−3.25 to 8.45)	3.65 (−6.28 to 5.60)	0.723	6.40 (−2.80 to 12.50)	0.70 (−4.70 to 10.20)	0.333	7.40 (2.18 to 14.03)	−6.60 (−8.60 to 2.13)	0.011^*^
	(*n*)	38	28		10	6		15	13		6	4	

* *p < 0.05 compared to the no-PFD control. Bold numbers denote statistical significance*.

Regarding the specific diseases, the improvement in FVC% was significantly higher in the SSc-PFD and IIM-PFD group with high baseline FVC% than patients with no-PFD: 6.60% (−1.23 to 11.50%) vs. 0.10% (−6.80 to 4.60%) (*p* = 0.047) and 6.30% (0.50 to 10.50%) vs. 1.10% (−3.30 to 6.80%) (*p* = 0.089), respectively. Among patients with RA, DLCo% showed a significant increase in the <70%-baseline-DLCo% subset given PFD compared to those not given PFD: 7.40% (2.18 to 14.03%) vs. −6.60% (−8.60 to 2.13%), *p* = 0.011. There was no significant improvement in DLCo% among patients with SSc after PFD treatment (Table 3).

### Analysis of PFD Dosage

In this study, a total of 56 patients in the PFD group received PFD for 24 weeks, and the dosage of PFD was adjusted according to the protocol. Four patients increased the dosage of PFD in the first 4 weeks, which then decreased to 400 mg/day due to their intolerance at 12 weeks and maintained up to 24 weeks. The others gradually increased the dosage up to the maximum tolerable dosage or to a maximum of 1,800 mg/day, including one patient taking a maximum tolerable dosage of 400 mg/day from the beginning. The mean daily dosage of PFD was 786.63 (682.87–896.5) mg/day, at the range of 400–1,800 mg/day (Supplementary Tables 4, 5).

When the PFD dosage was adjusted to 800 mg/day at 24 weeks, we found no differences in the change in PF or the reduction dosage of GC between high- and low-dosage-PFD subsets (Supplementary Table 6). There were no differences in the dosage of PFD across the PFD groups of the 4 diseases.

### Multiple Linear Regression Model for the Change in PF

Multiple linear regression analysis was used to identify the influencing factors of the change in FVC% and DLCo%. The basic clinical characteristics of patients with CTD-ILD, such as age, sex, body mass index (BMI), smoking history, disease duration, baseline FVC% < 70%, baseline DLCo% < 70%, dyspnea after activity, laboratory results, and UIP tendency on imaging, were first included. It was found that the baseline FVC% < 70% significantly affected the change in FVC%, and the baseline DLCo% < 70% affected the change in DLCo%. The therapeutic regimen was tested in another model, which included the average dosages of GC, HCQ, IS, and PFD as variables. The data indicated that PFD and high-dosage GC were positive factors for increased FVC%, and only PFD was significant in terms of DLCo% improvement (Supplementary Tables 7–10). In the pooled analysis, all factors affecting the changes in FVC% and DLCo% with *p* < 0.100 and the basic clinical index were included in the regression model, and baseline FVC% < 70% and PFD were found as positive factors influencing the changes in FVC% and DLCo% in CTD-ILD (Tables 4A,B).

**Table 4:** Multiple linear regression analysis of the change in PF in patients with CTD-ILD: multiple linear regression analysis of the change (A) in FVC% and (B) in DLCo%.

**Variables**	**Hazard Ratio**	**95% CI**		***p* value**
		**Lower**	**Upper**	
**(A) Multiple linear regression analysis of the change in FVC%**				
**Diseases**				
SSc	Ref	Ref	Ref	Ref
IIM	0.85	−4.52	6.23	0.753
RA	−2.68	−9.28	3.91	0.422
Other CTDs	−2.67	−9.82	4.48	0.461
Baseline FVC<70%	5.88	0.40	11.37	**0.036**
Glucocorticoid average dosage	0.11	−0.09	0.32	0.272
Pirfenidone	4.56	0.38	8.75	**0.033**
**(B) Multiple linear regression analysis of the change in DLCo%**				
**Diseases**				
SSc	Ref	Ref	Ref	Ref
IIM	2.35	−2.85	7.56	0.372
RA	−1.32	−8.19	5.54	0.703
Other CTDs	−2.09	−9.56	5.39	0.581
BMI	−0.27	−0.93	0.40	0.427
Baseline FVC<70%	6.81	1.28	12.33	**0.016**
Baseline DLCo<70%	−0.44	−5.11	4.22	0.850
Pirfenidone	4.37	0.02	8.72	**0.049**

*Bold numbers denote statistical significance*.

### AEs in the Study

The AEs that occurred during the study period are summarized in Table 5. A total of 30 AEs occurred in 21 patients in the PFD group compared with 22 AEs in 15 patients in the control group (32.80 vs. 20.83%, *p* = 0.124). The AEs that were more often found in the PFD group than the control group were gastrointestinal events, including abdominal distension, gastroesophageal reflux, and diarrhea (*p* = 0.067). All these AEs were mild to moderate, reversible, and without clinical sequelae. Respiratory infections were equal in both PFD and control patients (10.64 vs. 9.72%). There were no significant differences in the occurrence of other AEs, such as skin infections, urinary infections, and rash.

**Table 5:** Comparison of adverse events in the PFD and control groups over 24 weeks.

**Adverse event**	**Pirfenidone (*n* = 64)**	**Control (*n* = 72)**
Subjects (%)	21 (32.80)	15 (20.83)
Respiratory infections (%)	7 (10.94)	7 (9.72)
Skin infections (%)	1 (1.56)	1 (1.39)
Urinary infections (%)	2 (3.13)	2 (2.78)
Other infections (%)	1 (1.56)	1 (1.39)
Abdominal distension (%)	3 (4.69)	2 (2.78)
Gastroesophageal reflux (%)	4 (6.25)	1 (1.39)
Diarrhea (%)	2 (3.13)	0 (0.00)
AST and/or ALT increase (%)	1 (1.56)	2 (2.78)
Creatinine increase (%)	1 (1.56)	0 (0.00)
Rash (%)	3 (4.69)	4 (5.56)
Oral ulcer (%)	1 (1.56)	0 (0.00)
Respiratory failure (%)	1 (1.56)	0 (0.00)
Mediastinal emphysema (%)	1 (1.56)	1 (1.39)
Dizziness (%)	1 (1.56)	0 (0.00)
Palpitations (%)	1 (1.56)	0 (0.00)
Cerebral thrombosis (%)	0 (0.00)	1 (1.39)

Treatment-emergent serious AEs occurred in 3 patients (4.69%) in the PFD group and 6 patients (8.33%) in the control group. In the PFD group, a 57-year-old patient with melanoma differentiation-related gene 5 (MDA5)-positive dermatomyositis died of respiratory failure, mediastinal emphysema, and respiratory infections; this individual was receiving 15 mg/day of GC, 10 mg/day of tofacitinib, and 900 mg/day of PFD at that time. Two patients were hospitalized with infections (mumps and respiratory infections). In the control group, 1 patient experienced cerebral thrombosis, 3 patients were hospitalized for rash, and 2 subjects were hospitalized for respiratory infections.

## Discussion

The efficacy of PFD in the treatment of IPF has been confirmed by several clinical trials, which have shown that PFD may delay the decline in FVC and increase the progression-free survival rate (10–12, 24, 25). However, the clinical indication of PFD is still limited to patients with IPF, and no large cohort of PFD-treated patients with CTD-ILD has been reported. Therefore, our prospective study aimed to observe the efficacy of PFD in CTD-ILD and identify the best-responding HRCT subtype and baseline PF index among PFD-treated patients with CTD-ILD.

As this was a real-world study initiated by an investigator, we could not obtain free PFD for a randomized study on CTD-ILD. In contrast, the inclusion criteria of the PF threshold changed gradually in several previous studies on PFD. In a randomized controlled trial published in 2020 assessing the efficacy and safety of PFD in SSc-ILD (26), patients were included who met the disease criteria and had an FVC% value <80%. The inclusion criteria of the RELIEF study (27), a phase 2b trial on PFD, included patients with progressive fibrotic ILD, not limited to IPF, with FVC% and DLCo% values <90%. In a retrospective study on interstitial pneumonia with autoimmune features (IPAF), patients using PFD were enrolled without an upper limit for FVC% or DLCo% (28), and the therapeutic effect was also good. Although the criteria for the use of antifibrosis agents have been updated often in recent years, the benefits of PFD against CTD-ILD seem more pronounced. Meanwhile, the respiratory symptoms of patients with CTD-ILD were not completely consistent with their manifestations on HRCT scans and the PF. Therefore, we prescribed PFD for some patients with obvious imaging progress or UIP subtypes regardless of their PF to observe whether the rapid PF decline could be held off by PFD.

For these reasons, the patients in the PFD group had a poorer PF than the controls at baseline, which caused the imbalance and deviation in PF and HRCT. The patients needed to be grouped by their specific diseases, HRCT subtypes, and PF levels to reduce confounding effects, which made the subgroups too small for analysis. However, when we combined similar cases into a general cluster, the amount of GC and type of IS agent were not different between the HRCT and baseline PF subgroups, with or without PFD, but varied widely between different CTD-ILDs. Therefore, we described the results in terms of different diseases to minimize bias.

Recent studies have reported that a definite UIP pattern or a possible UIP pattern characterized by honeycomb and/or grid shadows in the ILD classification often demonstrates a poor prognosis (23). Therefore, we classified patients with a definite UIP pattern or a possible UIP pattern on HRCT as having a UIP tendency and found the IIM-non-UIP and RA-non-UIP tendency subtypes showed superior therapeutic effects of PFD. Interestingly, the change in FVC% in the SSc-UIP tendency subtype with PFD was higher than that in the control, a finding that needs to be supported by more data in a large number of patients with SSc-UIP tendency. Based on these results, we speculate that the PFD response could be influenced by both the imaging subtype and background disease.

To exclude the effect of differences in baseline PF between the PFD and control groups, we stratified the patients around a 70% baseline FVC% or DLCo% based on the phase III trial conducted in Japan (29). In general, the improvement in FVC% of the PFD group was higher than that of the control group regardless of the baseline value, but it was significantly greater in high-baseline-FVC% PFD-treated patients with SSc and IIM and low-baseline-DLCo% patients with RA.

We were delighted to find that the patients with CTD-ILD responded to PFD very well. The improvement in FVC% or DLCo% of the PFD group was more significant than that of the control after 24 weeks of treatment even though the PFD-treated patients were more likely to have a poorer PF and/or to have a UIP tendency at baseline. Multiple linear regression analysis also showed that baseline FVC% < 70% and PFD acted as positive factors for the changes in FVC% and DLCo% in CTD-ILD. As a result, patients with IIM and RA, with a non-UIP tendency and a lower PF at baseline, would most likely benefit from PFD.

In recent years, it has been noted that patients with IIM positive for anti MDA5 antibody usually have rapidly progressive interstitial lung disease (RP-ILD) and a poor prognosis. In this study, we enrolled 51 patients with IIM, including 20 MDA5-positive patients (10 in the PFD group and 10 in the control group) and 13 ARS-positive patients (6 in the PFD group and 7 in the control group) (Supplementary Table 2), and found that PFD improved the FVC% of the patients with IIM, especially the MDA5-positive patients. In the clinic, some of the MDA5-positive patients with IIM-ILD progressed too quickly to take antifibrosis agents, even with the help of a ventilator. Thus, the MDA5-positive patients in this study had an average baseline FVC% of 76.36 ± 19.95%, which was not as poor as expected, and showed a better response to PFD than other patients. In contrast, most of the patients had relatively stable disease activity during the observation, and there were no significant differences in rash, limb weakness, and hoarseness or dysphagia (Supplementary Table 11).

In 2015, the first multicenter study on PFD treatment in Chinese patients with IPF confirmed that PFD improved PF after 24 weeks (−0.08 ± 0.20 L vs.−0.22 ± 0.29 L) (30) and opened the door to PFD for IPF treatment in our country. A number of studies (13, 14, 26, 31) have found a beneficial effect of PFD in CTD-ILD, but the sample sizes have been too small. At present, PFD has been tested in randomized-controlled clinical studies in patients with different CTDs (SLSIII, NCT03221257; Trial 1, NCT02808871) (32), but the results have not yet been released.

Recently, a retrospective study at Tongji University (28) reported the effect of PFD on 184 patients with IPAF. The volume of FVC in the PFD group (*n* = 81) in this study was increased by 0.0390 L/year compared with a decrease of 0.0769 L/year in the control group (*n* = 103). The researchers concluded that PFD (600–1,800 mg/day) can improve FVC% and can help to reduce the GC dosage in patients with IPAF. In addition, the median dosage of PFD in that work was 1,492 mg/day.

In this study, a large proportion of patients took 800–1,200 mg/day PFD at 24 weeks, and the mean daily dosage of PFD during the 24-week period was 786.63 (682.87–896.5) mg/day, which was also lower than the standard dosage (Supplementary Tables 4, 5). Despite the inability to reach the full dosage of 1,800 mg/day due to individual tolerance, PFD was still effective in this study. Therefore, we may conclude that the tolerated dosage of PFD in Chinese patients with CTD-ILD is 800–1,200 mg/day, and the use of GC and IS may be associated with the low dosage of PFD. As there were no differences between the high- and low-dosage PFD groups (Supplementary Table 6), it will be necessary to monitor the blood concentration of PFD to determine its appropriate dosage in view of the interaction effect between GC, IS, and PFD.

As a prospective study, this study revealed the superiority of PF improvement by PFD. This benefit may be related to (1) the characteristic of the CTD background: patients with CTD-ILD have more inflammatory exudative lesions on HRCT scans, indicating conditions such as NSIP and OP, which could be absorbed in the short term; and (2) GC and IS background: PFD was used in combination with GC and IS, which helped in the recovery from ILD, especially the NSIP subtype. Although there were no significant differences in GC dosage and no IS changes in this study (Supplementary Tables 12–14), and all the patients demonstrated stabilization of the underlying disease (Supplementary Tables 2, 3), strictly paired clinical studies with large samples are needed.

Our study had some limitations. First, it was a single-center study with a limited sample size of each distinct CTD-ILD group. Due to the small numbers, there were a few differences in baseline characteristics, such as PF and HRCT features, between the study groups, and some of these factors cannot be ruled out as confounding factors. Simultaneously, masking of the differential effects of PFD due to treatment bias (GC and IS) of the underlying diseases cannot be excluded. Another shortcoming of this study was the lack of randomized control arms as the grouping in the study was partly determined by the patients based on their finances, insurance, and the self-assessment of their illness. Due to the high price of PFD, many real-world patients decide to add PFD when their disease becomes serious. Therefore, the patients in the PFD group in this study tended to have a poorer PF, and we could not exclude this baseline PF difference as a confounder. Moreover, the duration of follow-up was short, so we plan to extend this observation to 96 weeks.

## Conclusion

The response of PF to PFD differs between several kinds of CTD-ILD. PFD has a favorable benefit/risk profile and represents a suitable treatment option for patients with SSc, IIM, and RA who have a non-UIP tendency and lower PF.

## Data Availability Statement

The original contributions presented in the study are included in the article/Supplementary Material, further inquiries can be directed to the corresponding author/s.

## Ethics Statement

The studies involving human participants were reviewed and approved by the Ethics Committee of Qilu Hospital, Cheeloo College of Medicine, Shandong University (KYLL-202008-014). The patients/participants provided their written informed consent to participate in this study.

## Author Contributions

JW and QS collected tissue samples, drafted the manuscript, and designed the tables and figures. XW and XQ participated in manuscript preparation. TZ contributed to the data analysis and read the manuscript critically. YC participated in the HRCT imaging assessment and read the manuscript critically. ZS read the manuscript critically. All authors approved the final version of the manuscript.

## Funding

This study was supported by ECCM Program of Clinical Research Center of Shandong University (No. 2021SDUCRCB010).

## Conflict of Interest

The authors declare that the research was conducted in the absence of any commercial or financial relationships that could be construed as a potential conflict of interest.

## Publisher's Note

All claims expressed in this article are solely those of the authors and do not necessarily represent those of their affiliated organizations, or those of the publisher, the editors and the reviewers. Any product that may be evaluated in this article, or claim that may be made by its manufacturer, is not guaranteed or endorsed by the publisher.
