# Supplementary material for: The Efficacy and Safety of Pirfenidone Combined With Immunosuppressant Therapy in Connective Tissue Disease-Associated Interstitial Lung Disease: A 24-Week Prospective Controlled Cohort Study
Source: Front Med (Lausanne). 2022 May 12;9:871861. doi: 10.3389/fmed.2022.871861 (PMC9135161; doi:10.3389/fmed.2022.871861)
Supplement: Supplementary file 1 [file Data_Sheet_1.docx]

Supplementary Material

**Supplementary Appendix S1:**

Patients were excluded according to the following exclusion criteria:

(1) Subjects with non-diffuse connective tissue disease or arthritis other than RA, such as ANCA-related vasculitis or psoriatic arthritis;

(2) Patients who have ILD with clear aetiology, such as HIV or GVHD;

(3) Combined viscera function significantly abnormal patient:

a. Liver: AST, ALT >1.3 ULN; Bilirubin >1.5 ULN; or previous diagnosis of viral hepatitis;

b. Kidney: creatinine clearance <30 mL/min;

c. Lung: airway obstruction (pre-bronchodilator FEV1/FVC <0.7); pleural effusion accounts for more than 20% of the pleural effusion; severe pulmonary infection or other clinically significant pulmonary abnormalities;

d. Cardiovascular: myocardial infarction within six months;

e. Gastrointestinal tract: active peptic ulcer or bleeding;

f. Blood system: severe anaemia, decreased white blood cells and platelets;

g. Nervous system: mental disorders; cerebral thrombosis events (including stroke and transient ischaemic attack) within the past 1 year;

(4) Comorbid poor-prognosis disease such as cancer or genetic diseases;

(5) Women who were pregnant or lactating or of childbearing age and could not ensure effective contraception;

(6) According to the researchers, exhibited evidence of alcohol or drug abuse;

(7) Hypersensitivity to glucocorticoid, immunosuppressive agents or PFD.

| **Supplementary Table 1.** The skin phenotypes of SSc-ILD patients | | |
| --- | --- | --- |
|  | Pirfenidone (n=14) | Control (n=16) |
| **Skin phenotype** |  |  |
| Limited cutaneous SSc (%) | 5 (35.70) | 5 (31.30) |
| Diffuse cutaneous SSc (%) | 1 (7.10) | 2 (12.50) |
| Overlapping SSc (%) | 1 (7.10) | 3 (18.80) |
| Scleroderma sine SSc (%) | 7 (50.00) | 6 (37.50) |
| Facial skin involvement (%) | 4 (28.60) | 4 (25.00) |

| **Supplementary Table 2.** The myositis antibodies and basic disease activity of IIM-ILD patients at baseline and 24 weeks | | |
| --- | --- | --- |
|  | Pirfenidone (n=25) | Control (n=26) |
| **Myositis antibody** |  |  |
| MSA | 20 (80.00） | 20 (76.92） |
| MDA5 | 10 (40.00) | 10 (38.50) |
| ARS | 6 (24.00) | 7 (26.90) |
| Other MSAs | 4 (16.00) | 3 (11.50) |
| MAA | 4 (16.00) | 4 (15.40) |
| All negative | 1 (4.00) | 2 (7.70) |
| **Disease activity** |  |  |
| Rash (%) | 4 (16.00） | 10 (38.50） |
| Limb weakness (%) | 10 (40.00） | 12 (46.20） |
| Hoarseness and/or dysphagia (%) | 4 (16.00） | 3 (11.50） |
| 0w MMT8 | 79.00 (75.00~80.00) | 80.00 (78.00~80.00) |
| 0w MYOACT | 0.15 (0.13~0.17) | 0.15 (0.13~0.16) |
| 24w MMT8 | 80.00 (78.50~80.00) | 80.00 (80.00~80.00) |
| 24w MYOACT | 0.13 (0.13~0.15) | 0.15 (0.12~0.15) |
| **MSA**, myositis-specific autoantibodies; **MDA5**, anti-melanoma differentiation-related gene 5; **ARS**, anti-aminoacyl-tRNA-synthetase antibodies, including Jo-1, PL7, PL12, EJ and OJ; **Other MSAs** included Mi-2, TIF1-γ, NXP-2, SAE-1 and SRP; **MAA**, myositis-associated autoantibodies, including Ro52, Ku, PM-Scl 75 and PM-Scl 100; **MMT8,** manual muscle Test 8; **MYOACT**, myositis disease activity assessment visual analogue scale. | | |

| **Supplementary Table 3.** The antibodies and disease activity of arthritis in RA-ILD patients at baseline and 24 weeks | | |
| --- | --- | --- |
|  | Pirfenidone (n=10) | Control (n=7) |
| RF positive (%) | 9 （90.00) | 6 （85.71) |
| CCP positive (%) | 9 （90.00) | 6 （85.71) |
| **Disease activity of arthritis** |  |  |
| 0w DAS28 | 3.67 (3.45~4.31) | 3.57 (3.04~5.74) |
| 0w CDAI | 6.80 (4.53~8.75) | 6.10 (3.20~23.40) |
| 0w SDAI | 7.90 (5.89~9.78) | 6.91 (3.48~24.02) |
| 0w HAQ | 0.10 (0.09~0.13) | 0.05 (0.05~0.35) |
| 24w DAS28 | 2.89 (2.45~3.27) | 3.39 (2.75~3.93) |
| 24w CDAI | 3.90 (2.55~5.10) | 3.30 (1.20~7.00) |
| 24w SDAI | 4.11 (2.90~6.48) | 3.82 (1.77~7.59) |
| 24w HAQ | 0.05 (0.00~0.10) | 0.05 (0.00~0.10) |
| **RF**, rheumatoid factor; **CCP**, anti-cyclic citrullinated peptide; **DAS28**, disease activity score in 28 joints; **CDAI**, clinical disease activity index; **SDAI**, simplified disease activity index; **HAQ,** health assessment questionnaire; | | |

| **Supplementary Table 4.** The number of patients using different doses of PDF in each CTD-ILD at 24 weeks | | | | | |
| --- | --- | --- | --- | --- | --- |
| PFD dose (%) | Total (n=56) | SSc (n=14) | IIM (n=25) | RA (n=10) | Other CTDs (n=7) |
| 400 mg/d (%) | 5 (8.93) | 2(14.29) | 3 (12.00) | 0 (0.00) | 0 (0.00) |
| 600 mg/d (%) | 7 (12.50) | 2 (14.29) | 2 (8.00) | 2 (20.00) | 1 (14.29) |
| 800 mg/d (%) | 8 (14.29) | 0 (0.00) | 5 (20.00) | 1 (10.00) | 2 (42.89) |
| 900 mg/d (%) | 22 (39.29) | 8 (57.14) | 9 (36.00) | 2 (20.00) | 3 (42.89) |
| 1200 mg/d (%) | 12 (21.43) | 2 (14.29) | 5 (20.00) | 4 (40.00) | 1 (14.29) |
| 1500 mg/d (%) | 1 (1.79) | 0 (0.00) | 1 (4.00) | 0 (0.00) | 0 (0.00) |
| 1800 mg/d (%) | 1 (1.79) | 0 (0.00) | 0 (0.00) | 1 (10.00) | 0 (0.00) |
| **Other CTDs**: include SLE, pSS and UCTD | | | | |  |

| **Supplementary Table 5.** The distribution of mean daily dose of PFD in each CTD-ILD during the 24 weeks | | | | | |
| --- | --- | --- | --- | --- | --- |
| Dose of PFD (%) | Total (n=56) | SSc (n=14) | IIM (n=25) | RA (n=10) | Other CTDs (n=7) |
| 400-599 mg/d (%) | 7 (12.50) | 3 (21.40) | 3 (12.00) | 1 (10.00) | 0 (0.00) |
| 600-799 mg/d (%) | 23 (41.10) | 5 (35.70) | 9 (36.00) | 5 (50.00) | 4 (57.10) |
| 800-999 mg/d (%) | 18 (32.10) | 6 (42.90) | 9 (36.00) | 1 (10.00) | 2 (28.60) |
| 1000-1199 mg/d (%) | 5 (8.90) | 0 (0.00) | 2 (8.00) | 2 (20.00) | 1 (14.30) |
| 1200-1399 mg/d (%) | 2 (3.60) | 0 (0.00) | 2 (8.00) | 0 (0.00) | 0 (0.00) |
| 1400-1599 mg/d (%) | 0 (0.00) | 0 (0.00) | 0 (0.00) | 0 (0.00) | 0 (0.00) |
| 1600-1799 mg/d (%) | 1 (1.80) | 0 (0.00) | 0 (0.00) | 1 (10.00) | 0 (0.00) |
| Mean daily dose of PDF (mg/d) | 786.63 (682.87~896.50) | 782.79 (612.50~853.02) | 800.00 (629.50~946.65) | 786.60 (720.13~1084.45) | 791.60 (776.00~900.00) |

| **Supplementary Table 6.** The baseline and changes of PF and GC dosage in different PFD dosage subsets. | | | | | | |
| --- | --- | --- | --- | --- | --- | --- |
|  | High PFD | Low PFD | Control group | *p* value | | |
|  | n=36 | n=20 | n=55 | H vs. C | L vs. C | H vs. L |
| Baseline FVC% | 83.89±17.33 | 75.12±15.60 | 93.81±18.49 | **0.010** | **0.000** | 0.076 |
| Baseline DLCo% | 64.30±13.28 | 57.04±12.32 | 70.08±17.04 | 0.079 | **0.002** | 0.094 |
| The change in FVC% | 4.10 (0.10~8.46) | 7.00 (0.50~11.90) | 1.00 (-2.80~5.70) | 0.057 | **0.006** | 0.135 |
| The change in DLCo% | 0.20 (-4.20~7.80) | 4.60 (-2.80~12.50) | -2.80 (-6.10~4.70) | 0.149 | **0.036** | 0.214 |
| Baseline GC dose | 15.00 (5.00~25.00) | 25.00 (7.50~45.00) | 15.00 (7.50~20.00) | 0.522 | 0.193 | 0.097 |
| 24w GC dose | 7.50（5.00~12.50) | 7.50（5.00~14.38) | 8.50（2.50~12.50) | 0.825 | 0.861 | 0.680 |
| Average GC dose | 11.38（3.66~17.07) | 12.40（6.31~22.71) | 11.46（7.42~15.31) | 0.577 | 0.324 | 0.204 |
| **PF**, pulmonary function; **High PFD (H)**, average dose PFD>800 mg/day; **Low PFD (L)**,average dose ≤800 mg/day; **C**: Control group; **GC,** glucocorticoid | | | | | | |

| **Supplementary Table 7.** Multiple linear regression analysis of the change in FVC% with baseline clinical data of CTD-ILD patients | | | | |
| --- | --- | --- | --- | --- |
| Variable | Hazard Ratio | 95% CI | | p value |
|  |  | Lower | Upper |  |
| Age | 0.18 | -0.04 | 0.40 | 0.103 |
| BMI | -0.28 | -1.08 | 0.52 | 0.485 |
| Male | -1.79 | -9.89 | 6.31 | 0.662 |
| Smoking history | -2.71 | -12.26 | 6.84 | 0.574 |
| Disease course | -0.01 | -0.05 | 0.03 | 0.759 |
| Baseline FVC＜70% | 10.39 | 3.34 | 17.45 | **0.004** |
| Baseline DLCo＜70% | -0.16 | -5.14 | 4.83 | 0.95 |
| Activity-related dyspnoea | 0.66 | -5.16 | 6.47 | 0.822 |
| Unusual physical sign | -2.57 | -8.02 | 2.89 | 0.353 |
| Baseline ESR | -0.02 | -0.14 | 0.10 | 0.725 |
| Baseline CRP | -0.15 | -0.52 | 0.23 | 0.434 |
| Baseline haemoglobin | 0.01 | -0.16 | 0.18 | 0.896 |
| Baseline albumin | -0.27 | -0.90 | 0.35 | 0.385 |
| UIP tendency | -0.78 | -5.92 | 4.36 | 0.765 |

| **Supplementary Table 8.** Multiple linear regression analysis of the change in FVC% with therapeutic regimen of CTD-ILD patients | | | | |
| --- | --- | --- | --- | --- |
| Variable | Hazard Ratio | 95% CI | | p value |
|  |  | Lower | Upper |  |
| GC average dose | 0.23 | 0.05 | 0.42 | **0.014** |
| HCQ | -0.24 | -5.16 | 4.69 | 0.925 |
| Immunosuppressive drugs |  |  |  |  |
| None | ref | ref | ref | ref |
| MMF | -3.79 | -11.20 | 3.62 | 0.313 |
| TAC | 1.07 | -6.40 | 8.54 | 0.777 |
| JAKi | 0.17 | -8.00 | 8.34 | 0.967 |
| Others | -4.63 | -13.77 | 4.50 | 0.317 |
| Pirfenidone | 5.38 | 1.01 | 9.74 | **0.016** |
| **GC**, glucocorticoid; **HCQ**, hydroxychloroquine; **MMF**, mycophenolate mofetil; **TAC**, tacrolimus; **JAKi**, JAK inhibitor; **Others**: other immunosuppressive drugs, including iguratimod, cyclophosphamide and cyclosporine. | | | | |

| **Supplementary Table 9.** Multiple linear regression analysis of the change in DLCo% with baseline clinical data of CTD-ILD patients | | | | |
| --- | --- | --- | --- | --- |
| Variable | Hazard Ratio | 95% CI | | p value |
|  |  | Lower | Upper |  |
| Age | 0.17 | -0.04 | 0.37 | 0.107 |
| BMI | 0.66 | -0.06 | 1.38 | 0.07 |
| Male | 4.72 | -2.61 | 12.05 | 0.204 |
| Smoking history | -6.04 | -14.58 | 2.50 | 0.164 |
| Disease course | 0.01 | -0.03 | 0.04 | 0.775 |
| Baseline FVC＜70% | 5.76 | -0.67 | 12.18 | **0.079** |
| Baseline DLCo＜70% | 4.68 | 0.13 | 9.23 | **0.044** |
| Activity-related dyspnoea | -1.89 | -7.10 | 3.33 | 0.474 |
| Unusual physical sign | -2.58 | -7.46 | 2.31 | 0.297 |
| Baseline haemoglobin | -0.02 | -0.12 | 0.09 | 0.753 |
| Baseline CRP | 0.17 | -0.17 | 0.50 | 0.326 |
| Baseline HB | -0.07 | -0.22 | 0.08 | 0.376 |
| Baseline albumin | 0.38 | -0.20 | 0.96 | 0.193 |
| UIP tendency | -1.90 | -6.58 | 2.79 | 0.423 |

| **Supplementary Table 10.** Multiple linear regression analysis of the change in DLCo% with therapeutic regimen of CTD-ILD patients | | | | |
| --- | --- | --- | --- | --- |
| Variable | Hazard Ratio | 95% CI | | p value |
|  |  | Lower | Upper |  |
| GC average dose | 0.03 | -0.15 | 0.20 | 0.758 |
| HCQ | 2.51 | -2.14 | 7.16 | 0.287 |
| Immunosuppressive drugs |  |  |  |  |
| None | ref | ref | ref | ref |
| MMF | 3.13 | -3.78 | 10.04 | 0.371 |
| TAC | 4.72 | -2.12 | 11.56 | 0.174 |
| JAKi | -1.53 | -9.07 | 6.01 | 0.688 |
| Others | -2.29 | -10.65 | 6.08 | 0.589 |
| Pirfenidone | 4.39 | 0.34 | 8.44 | **0.034** |
| **GC**, glucocorticoid; **HCQ**, hydroxychloroquine; **MMF**, mycophenolate mofetil; **TAC**, tacrolimus; **JAKi**, JAK inhibitor; **Others**: other immunosuppressive drugs, including iguratimod, cyclophosphamide and cyclosporine. | | | | |

| **Supplementary Table 11.** The PF, HRCT imaging, basic disease activity and therapy regimen among IIM-ILD patients with different myositis antibodies at baseline and 24 weeks | | | | | | | | | | | | | | |
| --- | --- | --- | --- | --- | --- | --- | --- | --- | --- | --- | --- | --- | --- | --- |
|  | MSA positive | | | | | | | |  | MAA positive | |  | All negative | |
|  | MDA5 positive | |  | ARS positive | |  | Other MSAs positive | |  |  |  |  |  |  |
|  | Pirfenidone (n=10) | Control (n=10) |  | Pirfenidone (n=6) | Control (n=7) |  | Pirfenidone (n=4) | Control (n=3) |  | Pirfenidone (n=4) | Control (n=4) |  | Pirfenidone (n=1) | Control (n=2) |
| 0w FVC% | 76.36±19.95* | 98.14±14.99 |  | 79.56±17.31 | 78.29±15.25 |  | 78.80±21.95 | 95.30±12.80 |  | 81.91±18.56 | 94.5±20.08 |  | 72.00 | 87.85±3.04 |
| 0w DLCo% | 63.08±14.91 | 75.10±14.23 |  | 66.48±11.77 | 63.86±18.59 |  | 64.83±18.01 | 81.43±14.61 |  | 65.33±5.88 | 76.53±6.59 |  | 57.10 | 72.50±7.78 |
| 24w FVC% | 85.85±20.49 | 96.26±21.65 |  | 92.12±17.15 | 81.51±18.66 |  | 86.00±19.53 | 96.07±18.56 |  | 89.58±16.63 | 99.10±16.49 |  | 73.10 | 93.50±6.22 |
| 24w DLCo% | 66.77±14.12 | 72.81±15.74 |  | 67.45±9.45 | 62.00±18.73 |  | 64.43±13.21 | 76.97±21.00 |  | 74.75±11.14 | 83.75±13.72 |  | 51.80 * | 72.80±1.27 |
| the change in FVC% | **9.70 (0.65~16.88) *** | -0.85 (-8.53~6.18) |  | 5.55 (-0.08~22.19) | 0.00 (-5.40~7.20) |  | 7.10 (-0.55~14.95) | 1.10 (-2.90~4.60) |  | 10.50 (5.30~10.69) | 4.05 (1.55~7.65) |  | 5.65 (-) | 1.10 (-) |
| the change in DLCo% | 2.00 (-9.35~16.48) | -1.25 (-3.85~5.63) |  | -0.05 (-4.90~6.58) | -5.40 (-12.60~12.70) |  | 5.50 (-8.60~7.80) | -2.80 (-8.35~0.25) |  | 5.50 (2.75~5.95) | 7.25 (-5.60~20.05) |  | 0.30 (-) | -5.30 (-) |
| Thoracic HRCT scan (%) |  |  |  |  |  |  |  |  |  |  |  |  |  |  |
| UIP | 2 (20.00) | 0 (0.00) |  | 0 (0.00) | 0 (0.00) |  | 2 (50.00) | 0 (0.00) |  | 1 (25.00) | 1 (25.00) |  | 0 (0.00) | 0 (0.00) |
| NSIP | 7 (70.00) | 9 (90.00) |  | 6 (100.00) | 6 (85.70) |  | 2 (50.00) | 3 (100.00) |  | 3 (75.00) | 3 (75.00) |  | 1（100.00） | 2（100.00） |
| OP | 1 (10.00) | 1 (10.00) |  | 0 (0.00) | 1 (14.30) |  | 0 (0.00) | 0 (0.00) |  | 0 (0.00) | 0 (0.00) |  | 0 (0.00) | 0 (0.00) |
| LIP | 0 (0.00) | 0 (0.00) |  | 0 (0.00) | 0 (0.00) |  | 0 (0.00) | 0 (0.00) |  | 0 (0.00) | 0 (0.00) |  | 0 (0.00) | 0 (0.00) |
| UIP tendency on HRCT (%) | 5 (50.00) | 1 (10.00) |  | 3 (50.00) | 2 (28.60) |  | 2 (50.00) | 0 (0.00) |  | 2 (50.00) | 1 (25.00) |  | 1（100.00） | 0 (0.00) |
| Rash (%) | 4 (40.00) | 5 (50.00) |  | 0 (0.00) | 1 (14.30) |  | 4 (100.00) | 3 (100.00) |  | 0 (0.00) | 3 (75.00) |  | 0 (0.00) | 1（50.00） |
| Limb weakness (%) | 5 (50.00) | 3 (30.00) |  | 2 (33.30) | 5 (71.40) |  | 1 (25.00) | 1 (33.30) |  | 2 (50.00) | 2 (50.00) |  | 0 (0.00) | 1（50.00） |
| Hoarseness and/or dysphagia (%) | 4 (40.00) | 2 (20.00) |  | 0 (0.00) | 0 (0.00) |  | 0 (0.00) | 0 (0.00) |  | 0 (0.00) | 0(0.00) |  | 0 (0.00) | 1（50.00） |
| 0w MMT8 | 79.50 (72.25~80.00) | 80.00 (78.75~80.00) |  | 79.00 (74.00~80.00) | 79.00 (78.00~80.00) |  | 79.50 (77.50~80.00) | 80.00 (73.00~80.00) |  | 78.00 (70.00~79.00) | 78.50 (75.50~80.00) |  | 79.00 (-) | 76.00 (-) |
| 0w MYOACT | 0.17 (0.15~0.22) | 0.15 (0.13~0.18) |  | 0.14 (0.13~0.16) | 0.13 (0.13~0.15) |  | 0.15 (0.12~0.15) | 0.15 (0.13~0.16) |  | 0.13 (0.13~0.15) | 0.15 (0.14~0.15) |  | 0.18 (-) | 0.15 (-) |
| 24w MMT8 | 80.00 (78.50~80.00) | 80.00 (80.00~80.00) |  | 80.00 (79.50~80.00) | 80.00 (79.00~80.00) |  | 79.50 (78.50~80.00) | 80.00 (76.00~80.00) |  | 80.00 (78.50~80.00) | 80.00 (80.00~80.00) |  | 78.00 (-) | 78.00 (-) |
| 24w MYOACT | 0.13 (0.13~0.17) | 0.14 (0.13~0.15) |  | 0.13 (0.13~0.14) | 0.13 (0.12~0.14) |  | 0.13 (0.11~0.15) | 0.13 (0.12~0.15) |  | 0.12 (0.12~0.14) | 0.13 (0.11~0.13) |  | 13.75 (-) | 0.14 (-) |
| 0w GC dose (mg/d prednisone) | 27.50 (7.31~41.25) | 15.00 (11.25~38.75) |  | 32.50 (19.38~56.25) | 50.00 (20.00~60.00) |  | 37.50 (10.00~60.00) | 10.00 (10.00~12.50) |  | 20.00 (12.50~20.00) | 16.25 (13.75~17.50) |  | 28.75 (-) | 25.00 (-) |
| 24w GC dose (mg/d prednisone) | 8.00 (5.00~13.75) | 12.50 (6.88~15.00) |  | 11.25 (6.88~25.00) | 15.00 (12.50~20.00) |  | 18.75 (5.63~42.50) | 7.50 (6.25~10.00) |  | 10.00 (8.75~12.50) | 7.50 (6.25~8.75) |  | 13.75 (-) | 12.50 (-) |
| Average GC dose (mg/d prednisone) | 13.27 (6.20~26.14) | 12.74 (10.94~19.88) |  | 15.40 (12.08~45.40) | **22.08 (14.50~35.40)#** |  | 29.27 (8.44~52.09) | 8.93 (8.06~10.40) |  | 13.25 (9.75~15.38) | 9.85 (8.47~12.19) |  | 20.75 (-) | 17.50 (-) |
| DMARDs (%) |  |  |  |  |  |  |  |  |  |  |  |  |  |  |
| None | 1 (10.00) | 1 (10.00) |  | 0 (0.00) | 1 (14.30) |  | 2 (50.00) | 0 (0.00) |  | 0 (0.00) | 0 (0.00) |  | 1（100.00） | 0 (0.00) |
| MMF | 2 (20.00) | 1 (10.00) |  | 3 (50.00) | 1 (14.30) |  | 1 (25.00) | 2 (66.70) |  | 2 (50.00) | 0 (0.00) |  | 0 (0.00) | 2（100.00） |
| TAC | 1 (10.00) | 6 (60.00) |  | 3 (50.00) | 4 (57.10) |  | 1 (25.00) | 1 (33.30) |  | 2 (50.00) | 4 (10.00) |  | 0 (0.00) | 0 (0.00) |
| JAKi | 4 (40.00) | 2 (20.00) |  | 0 (0.00) | 0 (0.00) |  | 0 (0.00) | 0 (0.00) |  | 0 (0.00) | 0 (0.00) |  | 0 (0.00) | 0 (0.00) |
| Others | 2 (20.00) | 0 (0.00) |  | 0 (0.00) | 1 (14.30) |  | 0 (0.00) | 0 (0.00) |  | 0 (0.00) | 0 (0.00) |  | 0 (0.00) | 0 (0.00) |
| **MSA**, myositis-specific autoantibodies; **MAA**, myositis-associated autoantibodies, including Ro52, Ku, PM-Scl 75 and PM-Scl 100; **MDA5,** anti-melanoma differentiation-related gene 5; **ARS**, anti-aminoacyl-tRNA-synthetase antibodies, including Jo-1, PL7, PL12, EJ and OJ; **Other MSAs**, including Mi-2, TIF1-γ, NXP-2, SAE-1 and SRP; **UIP**, usual interstitial pneumonia; **NSIP**, nonspecific interstitial pneumonia; **LIP**, lymphocytic interstitial pneumonia; **OP**, organizing pneumonia; **UIP tendency on HRCT**: included definite UIP pattern and probable UIP pattern expressed by reticulation and honeycombing; **MMT8,** manual muscle Test 8; **MYOACT,** myositis disease activity assessment visual analogue scale; **GC**, glucocorticoid; **DMARDs**, disease-modifying antirheumatic drugs; **MMF**, mycophenolate mofetil; **TAC**, tacrolimus; **JAKi,** JAK inhibitor; **Others**: other immunosuppressive drugs, including cyclophosphamide and cyclosporine. **p* < 0.05 compared to the control group. # *p* < 0.05 compared to the other patients with different myositis antibodies in the control group. | | | | | | | | | | | | | | |

| **Supplementary Table 12.** The GC dosage in both PFD and control group of CTD-ILD patients.  The change in FVC% and DLCo% among different baseline PF subsets of the 3 CTD-ILD groups, with/without PFD. | | | | | | | | | | | | | | |
| --- | --- | --- | --- | --- | --- | --- | --- | --- | --- | --- | --- | --- | --- | --- |
|  | Total | |  | SSc-ILD | |  | IIM-ILD | |  | RA-ILD | |  | Other CTDs-ILD | |
|  | PFD (n=56) | Control (n=55) |  | PFD (n=14) | Control (n=16) |  | PFD (n=25) | Control (n=26) |  | PFD (n=10) | Control (n=7) |  | PFD (n=7) | Control (n=6) |
| Baseline GC dose | 15.00 (5.00~30.00) | 15.00 (7.50~20.00) |  | 6.25 (3.75~18.75) | 8.75 (5.00~14.38) |  | 25.00 (15.00~42.50) | 16.25 (12.50~50.00) |  | 12.50 (6.25~22.50) | 10.00 (7.50~20.00) |  | 5.00 (2.50~10.00) | 17.50 (2.13~30.00) |
| 24w GC dose | 7.50 (5.00~12.50) | 7.50 (5.00~12.50) |  | 5.00 (2.50~10.00) | 5.00 (2.50~6.88) |  | 10.00 (7.50~16.25) | 11.25 (7.50~15.00) |  | 11.25 (4.38~13.13) | 7.50 (5.00~12.50) |  | 5.00 (2.50~10.00) | 10.00 (2.23~10.63) |
| Average GC dose | 11.98 (5.00~18.90) | 15.00 (5.00~25.00) |  | 5.63 (2.66~14.58) | 7.50 (5.00~9.64) |  | 14.17 (11.64~26.18) | 13.21 (9.73~22.19) |  | 11.69 (4.56~18.65) | 11.67 (5.00~16.38) |  | 5.00 (2.50~11.96) | 14.59 (2.23~17.22) |

| **Supplementary Table 13.** The GC doses in the different HRCT subtypes of the 3 CTD-ILD groups | | | | | | | | | |
| --- | --- | --- | --- | --- | --- | --- | --- | --- | --- |
| Item | Category | SSc-ILD | |  | IIM-ILD | |  | RA-ILD | |
|  | HRCT | PFD | Control |  | PFD | Control |  | PFD | Control |
| Baseline GC dose | UIP tendency | 12.50 (2.81~45.00) | 7.50 (0.00~8.75) |  | 30.00 (18.75~42.50) | 17.50 (13.13~46.25) |  | 5.00 (0.63~9.38) | 15.00 (7.50~20.00) |
|  | N-UIP tendency | 5.00 (4.69~8.75) | 10.00 (5.00~15.00) |  | 22.50 (9.38~53.75) | 16.25 (11.88~50.00) |  | 20.00 (13.75~37.50) | 6.25 (2.50~10.00) |
| 24w GC dose | UIP tendency | 7.50 (2.50~13.75) | 5.00 (5.00~7.50) |  | 12.50 (8.00~16.25) | 13.75 (10.63~16.88) |  | 3.75 (0.63~6.88) | 7.50 (3.75~10.00) |
|  | N-UIP tendency | 3.75 (1.80~6.25) | 5.00 (2.50~6.25) |  | 7.50 (5.62~18.75) | 10.00 (7.50~15.00) |  | 12.50 (13.75~37.50) | 6.25 (2.50~10.00) |
| Average GC dose | UIP tendency | 10.31 (2.86~21.81) | 6.67 (0.00~8.75) |  | 17.50 (13.44~26.18) | 14.38 (11.33~20.62) |  | 3.88 (0.63~7.56) | 14.40 (6.25~16.94) |
|  | N-UIP tendency | 4.20 (2.03~7.53) | 7.50 (5.00~9.55) |  | 12.40 (7.73~37.3) | 13.21 (8.89~25.38) |  | 15.75 (11.84~25.77) | 7.09 (2.50~11.67) |

| **Supplementary Table 14.** The GC doses in the different baseline PF subsets of the 3 CTD-ILD groups | | | | | | | | | |
| --- | --- | --- | --- | --- | --- | --- | --- | --- | --- |
| Item | Category | SSc-ILD | |  | IIM-ILD | |  | RA-ILD | |
|  | Baseline PF | PFD | Control |  | PFD | Control |  | PFD | Control |
| Baseline GC dose | FVC%≥70% | 5.00 (3.75~15.00) | 10.00 (5.00~12.50) |  | 25.00 (7.50~35.00) | 15.00 (11.25~30.00) |  | 10.00 (3.75~20.00) | 10.00 (7.50~20.00) |
|  | FVC%˂70% | 12.50 (5.00~31.25) | - |  | 30.00 (15.00~52.50) | 55.00 (15.00~65.00) |  | 37.50 (15.00~60.00) | - |
| 24w GC dose | FVC%≥70% | 2.50 (2.50~7.50) | 5.00 (2.50~7.50) |  | 10.00 (5.00~12.50) | 10.00 (5.00~12.50) |  | 8.75 (3.13~12.50) | 7.50 (5.00~12.50) |
|  | FVC%˂70% | 10.00 (5.00~12.50) | - |  | 12.50 (7.50~35.00) | 15.00 (10.00~16.25) |  | 13.75 (12.50~15.00) | - |
| Average GC dose | FVC%≥70% | 3.93 (2.50~12.50) | 7.50 (5.00~9.72) |  | 13.63 (6.50~19.58) | 12.50 (8.84~17.63) |  | 10.17 (3.19~16.63) | 11.67 (5.00~16.35) |
|  | FVC%˂70% | 11.36 (5.00~19.43) | - |  | 21.80 (11.76~43.50) | 22.08 (13.87~39.35) |  | 22.87 (11.37~34.37) | - |
